# Successful perioperative management of a patient with erythropoietin-producing uterine myoma

**DOI:** 10.1186/s40981-018-0185-y

**Published:** 2018-06-18

**Authors:** Masato Kobayashi, Masahiko Akatsu, Yoshihisa Fujita, Koichi Nishikawa

**Affiliations:** 1 Department of Anesthesiology, Iwaki Kyoritsu General Hospital, 16 Kusehara, Mimaya-machi, Uchigo, Iwaki, Fukushima Prefecture 9738555 Japan; 2 Department of Disaster and Comprehensive Medicine, Fukushima Medical University, Fukushima, Fukushima Prefecture 9601295 Japan

**Keywords:** Erythropoietin-producing uterine myoma, Polycythemia, Polycythemia vera, Thrombosis, Phlebotomy, Isovolemic hemodilution, Fondaparinux

## Abstract

**Background:**

Erythropoietin-producing uterine myoma can cause various complications such as arterial or venous thrombosis and bleeding. Therefore, caution is required in the anesthetic management of affected patients.

**Case presentation:**

A 57-year-old female was suspected to have an erythropoietin-producing uterine myoma and was scheduled to undergo an abdominal total hysterectomy and bilateral salpingo-oophorectomy. Preoperative levels of hemoglobin and erythropoietin were 21.9 g/dl (normal 11.5–15 g/dl) and 23.2 IU/ml (normal 4.2–23.7 IU/ml), respectively. Preoperative phlebotomy and isovolemic hemodilution were performed to prevent arterial and venous thrombosis, following previous evidence that a hemoglobin level < 16 g/dl reduces the occurrence of polycythemia vera-related complications. Fondaparinux 2.5 mg was subcutaneously injected once daily after the operation, resulting in a good perioperative course without major complications.

**Conclusion:**

Herein, we have described a successful perioperative management of a patient with erythropoietin-producing uterine myoma. Our findings in this case suggest that this combination of antithrombotic therapies can facilitate anesthetic management of patients with this disease.

## Background

Polycythemia may cause arterial and venous thrombosis [1, 2]. While some reports are available regarding the anesthetic management of polycythemia vera [3–6], to the best of our knowledge, there are no previous reports regarding the anesthetic management of erythropoietin-producing uterine myoma. Herein, we report the successful administration of general anesthesia to a patient with erythropoietin-producing uterine myoma.

## Case presentation

**Fig. 1:**
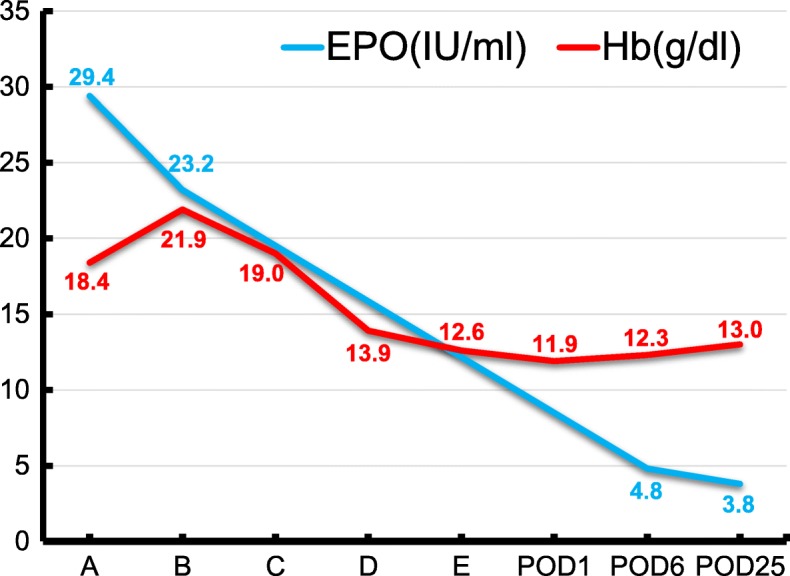
Changes in hemoglobin and erythropoietin levels. A, 9 years prior to reference to our hospital; B, at the time of the reference to our hospital; C, after phlebotomy; D, shortly before the operation; E, shortly after the operation, EPO, erythropoietin; POD, postoperative day

A 57-year-old female (body height 156 cm; body weight 64 kg) was referred to our hospital due to abdominal pain caused by a large uterine myoma. Nine years prior, she was diagnosed with polycythemia and an increased erythropoietin level (Fig. 1), although she was asymptomatic. At that time, the erythropoietin level soon began decreasing slightly without medication, and thus, the follow-up was completed. However, at the time of admission to our hospital, the patient’s blood test results had worsened. Although she did not report any symptoms other than abdominal pain and her activity level was not impeded, blood tests showed a relatively high level of erythropoietin and a remarkably high level of hemoglobin. Levels of hemoglobin and erythropoietin were 21.9 g/dl (normal 11.5–15 g/dl) and 23.2 IU/ml (normal 4.2–23.7 IU/ml), respectively (Fig. 1). Magnetic resonance imaging revealed a large uterine myoma measuring 25 cm in diameter. Therefore, she was suspected to have an erythropoietin-producing uterine myoma. There were no apparent symptoms of arterial or venous thrombosis or pulmonary embolism, which were ruled out by contrast computed tomography. Platelet count, coagulation test results, fibrinogen levels, and D-dimer levels were within normal ranges.

Prior to abdominal total hysterectomy and bilateral salpingo-oophorectomy, phlebotomy was scheduled to treat polycythemia; this reduced the risk of arterial and venous thrombosis. The patient was phlebotomized, 300 ml once a week, for up to 3 weeks without any complications. Despite the phlebotomy, hemoglobin levels remained high (Fig. 1); thus, isovolemic hemodilution was planned to be performed immediately following anesthesia induction.

Following placement of an epidural catheter into the epidural space at Th12/L1, general anesthesia was induced with 120 mg propofol, 0.1 mg fentanyl, and 50 mg rocuronium; it was maintained with 1.5% sevoflurane, 0.25 μg/kg/min remifentanil, and 10 mg rocuronium per 30 min. Electrocardiogram, bispectral index, end-tidal CO_2_, body temperature, and SpO2 were monitored during the surgery. Following induction of general anesthesia, an arterial 22 G catheter was placed in the radial artery, from which approximately 800 ml of blood was collected over 45 min while an equal amount of third-generation 6% hydroxyethyl starch (HES) 130/0.4/9 was infused from a peripheral venous 18 G catheter. As a result, the hemoglobin level dropped to 13.9 g/dl (Fig. 1). The surgery was performed with a total blood loss of 285 ml. During surgery, the infusion mainly comprised acetic acid Ringer’s solution and HES 130/0.4/9; the total infusion volume was 3600 ml. Determination of the infusion volume was based on cardiac and stroke volume indexes, measured with a FloTrac™/Vigileo™ system (Edwards Lifesciences, Irvine, CA, USA; SVV_FloTrac_). The patient’s urine volume was 590 ml. At the end of the surgery, the hemoglobin level was within the normal range (Fig. 1); thus, transfusion of autologous blood was not needed. Shortly after the end of the surgery, the trachea was uneventfully extubated, and the patient was transferred to the high care unit.

**Fig. 2:**
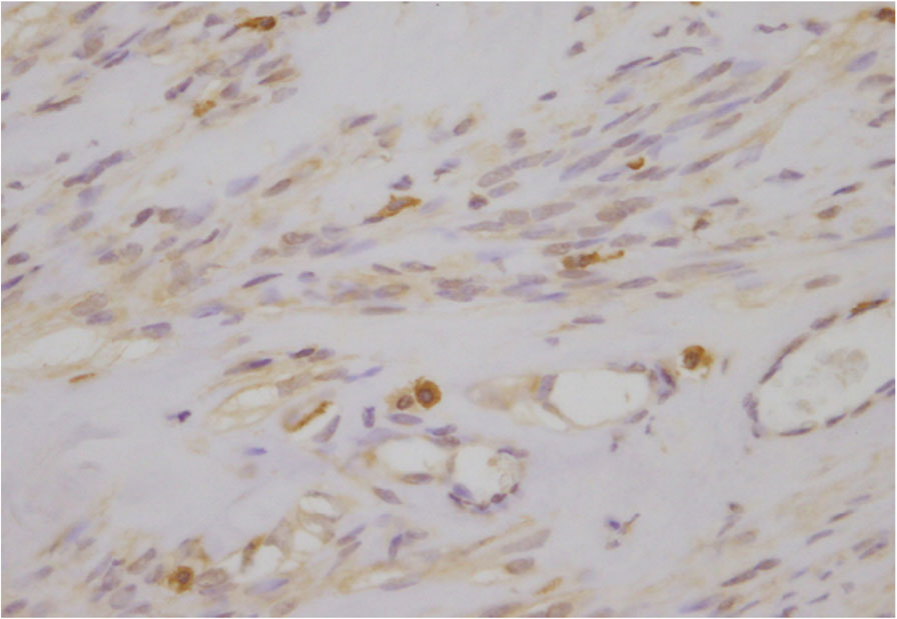
Pathological examination. Immunohistochemistry showed the presence of erythropoietin in the cells of the uterine myoma. Anti-erythropoietin polyclonal antibody stains the cytoplasm of smooth muscle cells brown. Magnification is × 40

On postoperative day (POD) 2, following removal of the epidural catheter, a daily subcutaneous injection of fondaparinux 2.5 mg was initiated and continued for 5 days to prevent deep vein thrombosis and pulmonary embolism. The postoperative course was uneventful, and there were no symptoms of thrombosis or bleeding. Continuous epidural analgesia with 0.25% levobupivacaine at a rate of 5 ml/h was performed postoperatively, and the patient did not report severe pain. Hemoglobin levels remained within the normal range, and the erythropoietin level dropped dramatically (Fig. 1). Pathological examination confirmed the production of erythropoietin from the tumor cell as well as the diagnosis of erythropoietin-producing uterine myoma (Fig. 2).

## Discussion

An erythropoietin-producing uterine myoma was first reported in 1953 by Thomson and Marson [7]. The prevalence of erythropoietin-producing tumors, which are mainly detected in renal cell carcinoma or hepatocellular carcinoma, is very low in secondary erythrocytosis; the prevalence of erythropoietin-producing uterine myoma is even lower, accounting for only 0.02–0.5% of all uterine myoma cases [8]. There are a few case reports of erythropoietin-producing uterine myoma investigated only from a gynecologic perspective [9, 10]. Polycythemia vera also occurs so rarely that there is currently insufficient evidence to establish a standard anesthetic management strategy. However, a few reports regarding anesthetic management of polycythemia vera can be found [3, 4], suggesting that specific measures are required for this disease. Therefore, we took several steps to prevent arterial and venous thrombosis, in accordance with some of those reports.

Phlebotomy has been used for many years and remains an efficient approach to dilute the concentration of red blood cells with few complications. It was previously reported that the use of phlebotomy to maintain the hemoglobin and hematocrit levels below 16 g/dl and 45%, respectively, reduces polycythemia vera-related complications [11, 12]. We performed phlebotomy in our patient, with a consequent decrease of the hemoglobin level. However, that decrease was insufficient to achieve the target value. With the assumption that hemoglobin and erythropoietin levels would remain low following myoma removal, we chose to proceed with the surgery, rather than repeat phlebotomy. Postponing the surgery would have extended the duration of polycythemia, increasing the risk of complications. Moreover, acute normovolemic hemodilution is helpful in cases that involve the potential for massive bleeding, as it reduces the essential amount of bleeding and avoids the risk of transfusion-related infections. In the present case, we used this method to dilute the concentration of red blood cells. It is ideal to use as thick a catheter as possible for collecting 800 ml of blood effectively without occlusion by thrombus. Although the thickest catheter we could place was 22 G because of the size of the artery, we were able to achieve the target hemoglobin level, as mentioned above.

The administration of low molecular weight or unfractionated heparin is also effective in reducing the risk of arterial and venous thrombosis associated with polycythemia vera, especially during the postoperative period [13]. In addition, anticoagulants including direct oral anticoagulants are recommended for the prevention of postoperative deep vein thrombosis rather than aspirin. In the present case, we administered fondaparinux, a selective inhibitor of factor Xa, instead of heparin. Notably, once daily injection of fondaparinux 2.5 mg has been demonstrated to reduce the relative risk of venous thrombosis in orthopedic surgeries by 50% compared to low molecular weight heparin [14]. In this case, favorable postoperative analgesia was achieved by means of epidural anesthesia. However, from the perspective of thrombosis prevention during the postoperative period, it may be reasonable to perform the ultrasound-guided transversus abdominis plane block and begin using heparin early in the postoperative period without the insertion of an epidural catheter.

Bleeding is reported as a major complication in polycythemia vera [15]. Patients with polycythemia vera can exhibit high platelet counts due to the influence of myeloproliferative disorder; bleeding tends to occur among patients with high platelet count [6, 16]. In contrast, erythropoietin-producing uterine myoma secretes only erythropoietin; therefore, it is not associated with changes in platelet count. Accordingly, our patient’s platelet count remained within the normal range. We also decided to place the epidural catheter into the epidural space for postoperative analgesia. LevGur and Levie suggested the possibility of bleeding in erythropoietin-producing uterine myoma, due to marked consumption of coagulation factors, which led to a deficiency of these factors [8]. This study further supports the use of fondaparinux. Fondaparinux is only associated with factor Xa inhibitory activity, whereas heparin also causes direct inactivation of thrombin. We believe that this characteristic of fondaparinux can lead to a lower incidence of bleeding compared with heparin. Mehta et al. reported 22% reduction in the relative risk of bleeding in the fondaparinux group compared to the unfractionated heparin group [17]. After careful consideration, the epidural catheter was successfully placed and removed without major bleeding, contributing to favorable analgesia.

### Conclusion

By using phlebotomy, isovolemic hemodilution, and subcutaneous fondaparinux, successful anesthetic management of a patient with erythropoietin-producing uterine myoma was performed. This successful management suggests the possible application of polycythemia vera-specific therapies to this disease, as well as to the prevention of arterial and venous thrombosis.

## Abbreviations

HES: Hydroxyethyl starch
POD: Postoperative day
